# Potential of cell tracking velocimetry as an economical and portable hematology analyzer

**DOI:** 10.1038/s41598-022-05654-5

**Published:** 2022-02-01

**Authors:** Jenifer Gómez-Pastora, Mitchell Weigand, James Kim, Andre F. Palmer, Mark Yazer, Payal C. Desai, Maciej Zborowski, Jeffrey J. Chalmers

**Affiliations:** 1 William G. Lowrie Department of Chemical and Biomolecular Engineering, The Ohio State University, 151 West Woodruff Avenue, Columbus, OH 43210 USA; 2 Department of Pathology, University of Pittsburgh, 3636 Blvd of the Allies, Pittsburgh, PA 15213 USA; 3 Division of Hematology, Department of Internal Medicine, The Ohio State University, Lincoln Tower - 1100-D, 1800 Cannon Drive, Columbus, OH 43210 USA; 4 Department of Biomedical Engineering, Cleveland Clinic, 9500 Euclid Avenue, Cleveland, OH 44195 USA

**Keywords:** Biophysics, Biotechnology, Cardiology, Diseases, Medical research, Engineering

## Abstract

Anemia and iron deficiency continue to be the most prevalent nutritional disorders in the world, affecting billions of people in both developed and developing countries. The initial diagnosis of anemia is typically based on several markers, including red blood cell (RBC) count, hematocrit and total hemoglobin. Using modern hematology analyzers, erythrocyte parameters such as mean corpuscular volume (MCV), mean corpuscular hemoglobin (MCH), etc. are also being used. However, most of these commercially available analyzers pose several disadvantages: they are expensive instruments that require significant bench space and are heavy enough to limit their use to a specific lab and lead to a delay in results, making them less practical as a point-of-care instrument that can be used for swift clinical evaluation. Thus, there is a need for a portable and economical hematology analyzer that can be used at the point of need. In this work, we evaluated the performance of a system referred to as the cell tracking velocimetry (CTV) to measure several hematological parameters from fresh human blood obtained from healthy donors and from sickle cell disease subjects. Our system, based on the paramagnetic behavior that deoxyhemoglobin or methemoglobin containing RBCs experience when suspended in water after applying a magnetic field, uses a combination of magnets and microfluidics and has the ability to track the movement of thousands of red cells in a short period of time. This allows us to measure not only traditional RBC indices but also novel parameters that are only available for analyzers that assess erythrocytes on a cell by cell basis. As such, we report, for the first time, the use of our CTV as a hematology analyzer that is able to measure MCV, MCH, mean corpuscular hemoglobin concentration (MCHC), red cell distribution width (RDW), the percentage of hypochromic cells (which is an indicator of insufficient marrow iron supply that reflects recent iron reduction), and the correlation coefficients between these metrics. Our initial results indicate that most of the parameters measured with CTV are within the normal range for healthy adults. Only the parameters related to the red cell volume (primarily MCV and RDW) were outside the normal range. We observed significant discrepancies between the MCV measured by our technology (and also by an automated cell counter) and the manual method that calculates MCV through the hematocrit obtained by packed cell volume, which are attributed to the artifacts of plasma trapping and cell shrinkage. While there may be limitations for measuring MCV, this device offers a novel point of care instrument to provide rapid RBC parameters such as iron stores that are otherwise not rapidly available to the clinician. Thus, our CTV is a promising technology with the potential to be employed as an accurate, economical, portable and fast hematology analyzer after applying instrument-specific reference ranges or correction factors.

## Introduction

Despite global progress achieved in medicine and science, anemia continues to be one of the most prevalent disorders. Anemia is defined by the World Health Organization (WHO) as a condition in which the hemoglobin (Hb) concentration in blood is lower than 130 g/L in men and lower than 110 g/L in pregnant women^1^. It affects more than 30% of the world’s population, of which more than 50% suffers from iron deficiency anemia (IDA)^2,3^. In fact, iron deficiency (ID) is the most widespread nutritional disorder in the world, afflicting between 2 and 4 billion people worldwide^4,5^. IDA is particularly prevalent for children and women in both developing and developed countries^6–9^. ID leads to the suppression of Hb synthesis, induces metabolic disorders, affects cognitive and motor development, and causes fatigue and decreased productivity. One of the most common causes to develop iron deficiency is blood loss; for example, iron deficiency anemia may be developed after blood donation if iron stores are limited^6^. In fact, 35% of the 9 million blood donors in the United States are estimated to be iron deficient^10^.

Iron deficiency/Iron deficiency anemia can be diagnosed by different biochemical analyses. Determination of red blood cell (RBC) count, hematocrit (Hct) and Hb are routine laboratory tests to determine the presence of anemia. Based on the work by Wintrobe in the early 1930s, RBC indices such as mean corpuscular volume (MCV), mean corpuscular hemoglobin concentration (MCHC) and mean corpuscular hemoglobin (MCH) were established to characterize the RBCs of anemic subjects^11^. These are valuable data for the diagnosis of ID or IDA and can be easily acquired by automated blood cell counters. In fact, modern analyzers such as ADVIA (Siemens Healthineers Inc.) uses a range of technologies such as flow cytometry, chemical reaction and spectral absorption reading to provide several indexes, such as reticulocyte count, (early index of ID, as reticulocytes exist in the circulation for only 1–2 days), hypochromic RBC count, (cells with a Hb concentration lower than 28 g/dL, an indicator of insufficient marrow iron supply that reflects recent iron reduction), etc.^6,11,12^.

Although these instruments are accurate, reproducible, and sometimes fast, the cost of the newest, high-volume analyzers is high and can vary from a single instrument of approximately $75,000 to an automated multiple-instrument system in excess of $200,000 dollars^13^. Also, these are large and heavy equipment, sacrificing portability; thus, samples collected off-site present a time delay in analyzing the sample, introducing risks of inaccuracy and contamination. Furthermore, they may require trained laboratory technicians, high blood volume requirements, and costly reagents for sample pretreatment^14^. Moreover, the automated hematology analyzers are not standardized among manufacturers due to patent issues, which makes necessary the establishment of instrument-specific reference ranges and clinical decision values^15,16^.

We have previously developed a system, referred to as the cell tracking velocimetry (CTV), to measure the mass of Hb in fresh RBCs on a cell by cell basis using principles from the pioneering work of Pauling and Coryell studying the magnetism of Hb in different chemical states^17,18^. CTV uses a combination of a microscope camera and a microfluidic channel within a well-defined magnetic energy gradient to track the movement of cells and particles under the direct influence of magnetic and gravitational fields^19–21^. Mathematically, the magnetically and gravitationally induced velocities, u_m_ and u_s_, can be described as follows:

1 $${\text{u}}_{{\text{m}}} = \frac{{\left( {\upchi _{{{\text{Cell}}}} -\upchi _{{{\text{Fluid}}}} } \right){\text{V}}_{{{\text{Cell}}}} }}{{3\uppi {\text{D}}_{{{\text{Cell}}}}\upeta }}{\text{S}}_{{\text{m}}}$$

2 $${\text{u}}_{{\text{s}}} = \frac{{\left( {\uprho _{{{\text{Cell}}}} -\uprho _{{{\text{Fluid}}}} } \right){\text{V}}_{{{\text{Cell}}}} }}{{3\uppi {\text{D}}_{{{\text{Cell}}}}\upeta }}{\text{g}}$$

where the subscripts cell and fluid refer to the cell and suspending fluid, χ is the magnetic susceptibility, ρ is the density (1,100 kg/m^3^), D and V are the diameter and volume of a cell (particle), η is the viscosity of the suspending fluid (9.7*10^–4^ Pa*s), and g is the acceleration due to gravity (9.8 m/s^2^). S_m_, the magnetic energy gradient, is defined by:

3 $${\text{S}}_{{\text{m}}} = \frac{{\left| {\nabla {\text{B}}^{2} } \right|}}{{2\upmu _{0} }}$$

where µ_0_ and B are the permeability of free space and the magnetic flux density at the cell location.

Rearranging Eq. (2) can yield the MCV, taking into account the disc shaped fresh RBC, as follows:

4 $${\text{D}}_{{{\text{RBC}}}} = \left[ {\frac{{1.349* {\text{ u}}_{{\text{s}}} *\upeta * 18}}{{{\Delta \rho }* {\text{ g}}}}} \right]^{0.5}$$

5 $${\text{MCV}}_{{{\text{CTV}}}} = {\text{h}}_{{{\text{RBC}}}} *\uppi *\left[ {\frac{{{\text{D}}_{{{\text{RBC}}}} }}{2}} \right]^{2}$$

where 1.349 is the sedimentation rate (u_s_) drag factor found by Zhbanov et al.^22^ and h_RBC_ is the average RBC thickness (2.25 µm). From the MCV distribution, the RBC distribution width (RDW_CTV_) can also be obtained.

Previously, the relationship between u_m_, u_s_, and the amount of hemoglobin in the RBC, accounting for volume, has been described in detail^23,24^:

6 $${\text{MCHC}}_{{{\text{CTV}}}} { } = \frac{{\left( {\frac{{{\text{u}}_{{\text{m}}} }}{{{\text{u}}_{{\text{s}}} }}} \right)\left( {{\Delta \rho }} \right)\left( {\frac{{\text{g}}}{{{\text{S}}_{{\text{m}}} }}} \right)}}{{\left( {\upchi _{{{\text{m}},{\text{metHb}}}} +\upchi _{{{\text{m}},{\text{globin}}}} -\upchi _{{{\text{H}}_{2} {\text{O}}}} } \right)*{\text{V}}_{{{\text{m}},{\text{Hb}}}} }}*{\text{MW}}_{{{\text{Hb}}}}$$

7 $${\text{MCH}}_{{{\text{CTV}}}} = \frac{{9*2^{0.5}\uppi }}{{{\text{S}}_{{\text{m}}} *\left( {\upchi _{{{\text{m}},{\text{metHb}}}} +\upchi _{{{\text{m}},{\text{globin}}}} -\upchi _{{{\text{H}}_{2} {\text{O}}}} } \right)*{\text{V}}_{{{\text{m}},{\text{Hb}}}} }}*\left[ {\frac{{\left( {1.23*{\text{u}}_{{\text{m}}} } \right)*\left( {1.23*{\text{u}}_{{\text{s}}} } \right)^{0.5} *\upeta ^{1.5} }}{{{\Delta \rho }^{0.5}* {\text{g}}^{0.5} }}} \right]*10^{3} *{\text{MW}}_{{{\text{Hb}}}}$$

where V_m,Hb_ = 48.23 L/mol is the molar volume of metHb, χ_m,globin_ = − 37,830 × 10^–9^ L/mol is the molar susceptibility of the globin chain, and χ_H2O_ = − 12.97 × 10^–9^ L/mol is the molar susceptibility of water. The molar susceptibility of the deoxyHb heme group is χ_m,deoxyHb_ = 50,890 × 10^–9^ L/mol, and that of metHb heme group is χ_m,metHb_ = 56,000 × 10^–9^ L/mol (all in CGS system of units). From these data, the percentage of RBCs with abnormal Hb concentration, such as hypochromic RBCs, which is a useful parameter for the detection of anemias, can be easily calculated (Hypo_CTV_).

Using these features, the CTV has the potential to be employed as a portable, low-cost hematology analyzer. In this work, we report for the first time the use of our CTV as a hematology analyzer able to measure both traditional RBC indices (MCV, MCHC and MCH) and novel indices such as the percentage of hypochromic RBCs and RDW, showing for the first time the capability of this technology to perform high precision flow cytometry analysis.

## Materials and methods

### Sample preparation

Thirty-one whole blood samples from 6 healthy donors and 15 blood samples obtained from 15 sickle cell disease (SCD) subjects were analyzed. Some of these SCD samples were collected after the subject received an exchange transfusion and others had not received a transfusion for at least 3 months prior to sample collection. All samples were collected following informed consent according to protocols approved by the Institutional Review Board (IRB) of The Ohio State University (protocol numbers 2021H0075 and 2018H0268). All experiments were performed in accordance with relevant guidelines and regulations. For the healthy blood donors, a total of approximately 8 mL of whole blood was drawn into a 10 mL collection tube containing EDTA anticoagulant. These samples were separated into two aliquots, each designated for whole blood analysis and RBC analysis. The RBC aliquots were further processed and washed in PBS to separate the RBCs from plasma (three times with 200× g centrifugation for 5 min). For the SCD subjects, either whole blood (from subjects not receiving transfusion therapy) or the apheresis waste (i.e. discarded RBCs) following a routine exchange transfusion (from subjects receiving a transfusion) were collected. The RBCs from the SCD samples, prior to their analysis via CTV, were processed and washed in PBS three times.

### Coulter counter analysis

All samples were introduced to our automated cell counter, B23005 Multisizer 4e Coulter Counter (CC, Beckman Coulter, CA), to measure the cell concentration as well as volume, MCV. The indicators measured with this instrument are denoted by a CC subscript (e.g., RBC count_CC_, MCV_CC_).

### Spectrophotometric analysis

Total hemoglobin, Hb, was measured only from the healthy whole blood samples by using the spectrophotometric method commonly employed in clinical laboratories, as described in our previous work^24^. Briefly, the samples obtained for spectrophotometric Hb concentration determination were diluted (4X) in deionized water in order to lyse the RBCs. The samples were allowed to sit for 30 min at 6 °C to ensure complete lysis. Then, the lysed cells were pelleted via tabletop centrifugation (2000× g). The concentration of Hb in the supernatant was assessed via OLIS Spectral-works (Olis, Inc., GA), where the diluted samples were further diluted to ensure that the absorbance around the Q bands (~ 540 nm and 575 nm) was between 0.1 and 1.0 for accuracy in readings according to Beer-Lambert’s law. Each sample was tested in triplicate and the mean and standard deviation of the absorbance were calculated. The Hb measured by this method is denoted by a “spec” subscript (Hb_spec_).

### CTV analysis

**Figure 1:**
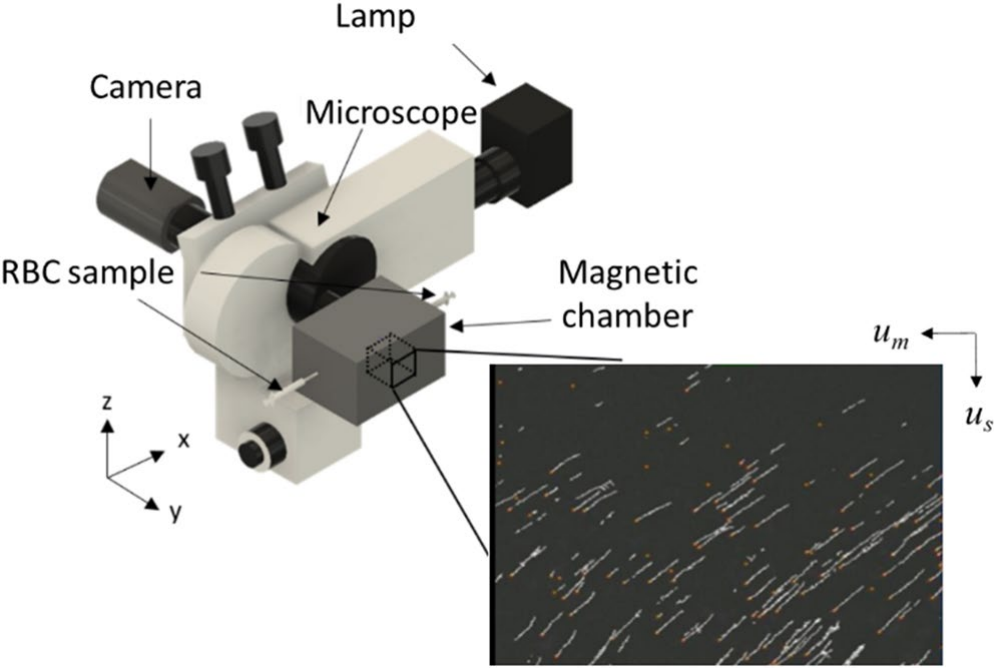
Schematic representation of the CTV instrument (adapted from Ref.^24^).

Both healthy RBCs and sickle RBCs were analyzed using CTV. The calibration and operating procedure for using CTV to magnetically characterize RBCs has been described in previous reports^24,25^. Supplementary Figures S1 and S2 report the working principle and the optimization of the CTV operation procedure. Briefly, RBCs were oxidized with a sodium nitrite (NaNO_2_) solution to turn them into methemoglobin containing RBCs (metHb-RBCs), then the metHb-RBCs were introduced into the CTV instrument at a concentration of 1 million cells/mL and the channels were sealed on both ends using Hamilton 1–1 valves (Hamilton Company, NV) to dampen any flow disturbances. After approximately 20 s, images of cell movement were captured (50 images at 1 s interval) and the captured images were further processed using an in-house analysis program that can convert the captured movement of the cells into magnetic and settling velocity of the cells. Figure 1 shows a schematic representation of the CTV instrument.

### Packed-cell volume

The conventional Hct test, also known as a packed-cell volume (PCV) test, was performed on healthy RBC samples by the micro-hematocrit centrifugation method. A hematocrit capillary tube (Drummond Scientific Company) was filled with whole blood and capped on one end with a clay sealant. Then, the tubes were centrifuged at 17,000× g for 5 min in a Sorvall Legend Micro 17 (Thermo Scientific), and the Hct was read from the capillary tube once aligned with a provided chart and the result was round to the nearest half percent. The Hct measured by this method is denoted by a PCV subscript (Hct_PCV_).

### Other estimations for erythrocyte indices

As stated before, the RBC indices are useful to determine if a subject is anemic, as well as to initially classify the anemia state. Hb, Hct and RBC count are usually employed to estimate three indices: MCV, MCHC and MCH. While these indices can be directly measured on CTV or on automated cell counters, we also estimate them from the Hb, RBC count and Hct values for comparison purposes. Thus, the average MCV, defined as the average volume of RBCs, and usually expressed in femtoliters (fL), can be obtained from the Hct (Hct_PCV_) and the RBC count (RBC count_CC_), as follows:

8 $${\text{MCV}}_{{{\text{ave}}}} = \frac{{{\text{Hct}}_{{{\text{PCV}}}} { }\left( {{\text{L}}/{\text{L}}} \right)}}{{{\text{RBC}}\;{\text{ count}}_{{{\text{CC}}}} { }\left( { \times { }10^{12} /{\text{L}}} \right)}}$$

On the other hand, the MCHC is the average concentration of hemoglobin in the RBCs, usually expressed in g/dL, and is calculated from the Hb (Hb_spec_) and Hct as follows:

9 $${\text{MCHC}}_{{{\text{ave}}}} = \frac{{{\text{Hb}}_{{{\text{spec}}}} { }\left( {{\text{g}}/{\text{dL}}} \right)}}{{{\text{Hct}}_{{{\text{PCV}}}} { }\left( {{\text{L}}/{\text{L}}} \right)}}$$

Finally, the MCH measures the average mass of hemoglobin in individual RBCs, usually expressed in pg, and is calculated from the Hb and RBC count as follows:

10 $${\text{MCH}}_{{{\text{ave}}}} = \frac{{{\text{Hb}}_{{{\text{spec}}}} { }\left( {{\text{g}}/{\text{dL}}} \right)}}{{{\text{RBC}}\;{\text{ count}}_{{{\text{CC}}}} { }\left( { \times { }10^{12} /{\text{L}}} \right)}}$$

These three estimated average indices (with “ave” subscripts) will be calculated in this work based on the Hct, Hb and RBC count provided by the traditional methods (Hct_PCV_, Hb_spec_ and RBC count_CC_) and compared with direct measurements taken from CTV and Coulter Counter.

## Results and discussion

Before analyzing abnormal sickle RBC samples, a total of 31 healthy RBC samples were collected and the following parameters; Hb concentration, Hct, RBC count, MCV, RDW, MCH, MCHC and Hypo, were measured by using a combination of different methods explained above. The results of the measurements for these healthy RBC samples are presented in Table 1. As expected, most of the test results show a healthy blood status of the donors with their respective parameters within the normal ranges^26^. However, some parameters related to the RBC volume (MCV, MCHC and RDW) and measured by CC or CTV are outside the normal range. These discrepancies are thoroughly analyzed and discussed in the following.

**Table 1:** Hb concentration, Hct, RBC count, MCV, RDW, MCH, MCHC and Hypo measured by different methods for 31 fresh healthy human blood samples.

Donor #	Gender	Age	Hb_spec_ (g/dL)	Hct_PCV_ (%)	RBC count_CC_ (10^6^ cells/μL)	MCV_ave_ (fL)	MCV_CC_ (fL)	RDW_CC_ (%)	MCV_CTV_ (fL)	RDW_CTV_ (%)	MCH _CTV_ (pgHb)	MCHC_CTV_ (g/dL)	Hypo_CTV_ (%)
1A	M	29	17.76	51	6.60	76.80	49.76	26	52.88	20	36.11	50.59	9
1B	M		7.05	48	5.94	79.59	49.20	30	47.62	22	26.19	44.31	9
1C	M		15.34	46	6.30	71.93	47.46	28	52.15	23	29.37	44.03	8
1D	M		17.70	51	4.97	103.20	47.65	30	47.65	24	25.46	43.13	13
1E	M		17.18	49	5.52	89.03	50.65	24	49.77	30	35.85	61.07	3
2A	F	31	15.15	46	5.70	80.80	54.97	26	51.68	22	38.48	58.89	10
2B	F		16.68	46	4.60	100.25	56.95	28	52.49	19	28.24	40.13	10
2C	F		15.78	45	5.59	80.55	56.56	24	49.95	26	29.34	48.98	15
2D	F		15.78	45	4.36	103.10	56.07	24	51.17	23	31.03	49.39	6
2E	F		14.92	45	6.39	70.46	53.53	23	50.40	24	26.64	46.13	5
2F	F		15.62	45	4.36	103.21	55.69	23	54.28	35	34.49	52.28	10
2G	F		15.62	45	4.38	102.65	57.08	22	52.64	34	37.43	61.75	26
2H	F		15.14	46	4.38	104.93	55.24	23	52.01	21	25.75	39.12	16
2I	F		17.28	48	4.38	109.49	55.51	22	51.15	23	34.10	51.14	5
3A	M	32	17.22	48	5.20	91.56	52.67	25	52.33	23	31.99	48.45	29
3B	M		18.77	50	5.65	90.32	53.74	25	47.86	20	27.96	46.35	5
3C	M		16.02	48	5.07	94.74	50.67	24	50.17	21	25.71	38.24	23
3D	M		17.47	50	4.40	110.88	55.17	22	49.92	22	30.52	49.85	5
3E	M		16.37	50	6.20	80.57	50.67	28	47.89	22	26.10	45.54	7
4A	F	24	15.26	44	5.30	83.03	55.32	24	50.43	23	30.62	47.37	5
4B	F		15.45	44	5.32	82.67	52.27	27	45.62	27	24.25	46.09	12
5A	M	60	15.26	43	4.85	89.60	52.66	27	50.52	26	27.56	43.01	12
5B	M		15.57	45	4.38	102.74	53.49	22	48.94	25	30.82	50.31	12
5C	M		15.98	45	4.59	98.11	52.68	22	50.28	21	43.75	66.04	7
5D	M		15.98	44	4.37	99.87	53.94	20	48.36	24	30.83	50.28	10
5E	M		15.13	45	4.74	94.90	53.87	25	52.02	22	23.04	33.27	29
6A	F	22	12.76	42	5.60	75.05	43.40	27	43.40	25	15.40	28.92	55
6B	F		12.90	43	4.96	86.77	44.56	24	45.60	19	41.67	74.10	3
6C	F		12.90	50	5.60	89.13	45.42	23	43.84	29	20.70	40.12	34
6D	F		12.96	42	4.87	86.24	44.64	24	52.18	19	24.38	35.60	22
6E	F		12.73	42	4.53	92.78	44.61	31	41.57	24	24.51	50.06	5
Average			15.35	46	5.13	91.13	51.81	25	49.57	24	29.62	47.89	14

### Mean corpuscular volume

**Figure 2:**
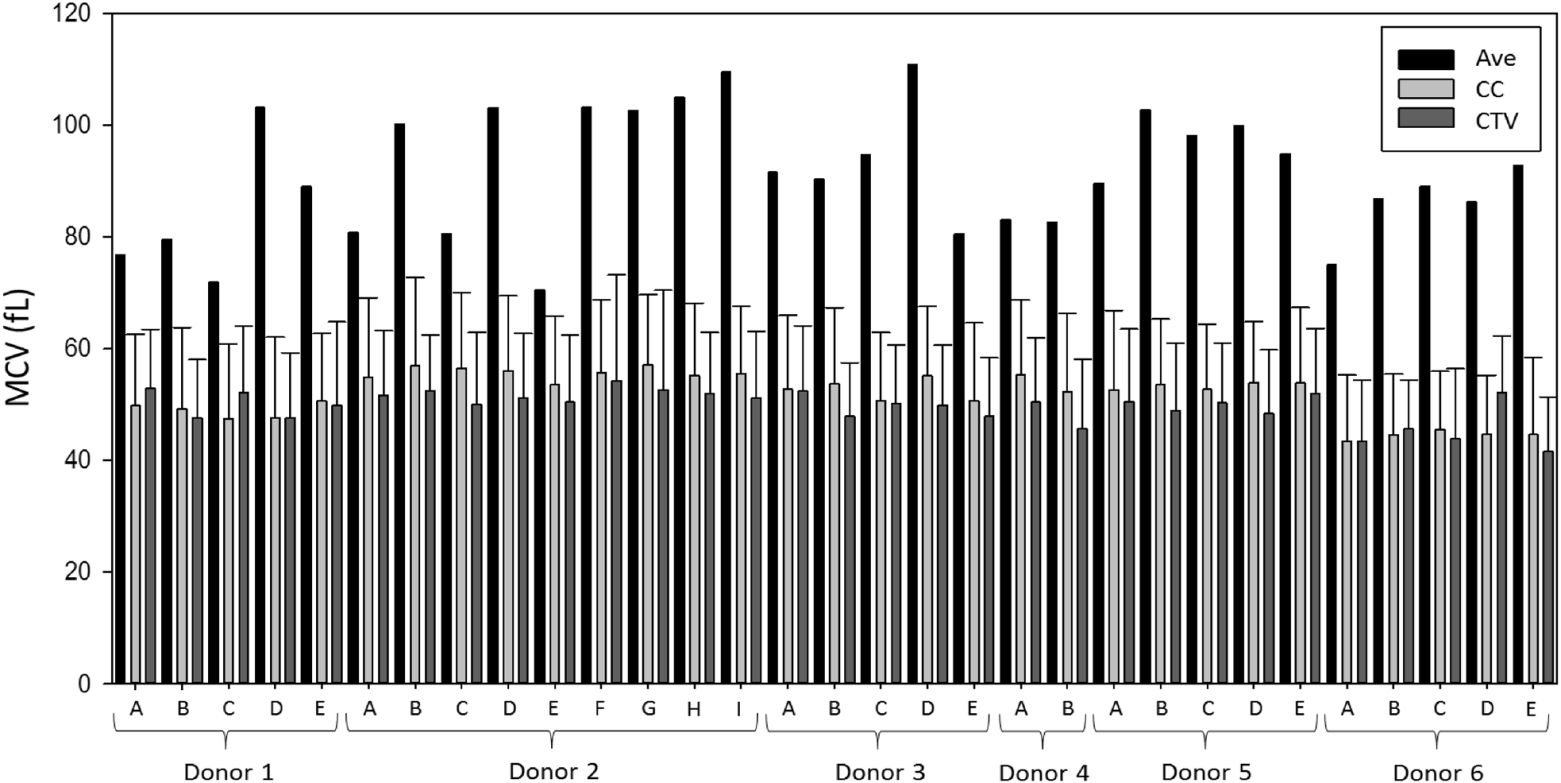
Comparison between the MCV_ave_ (calculated from Hct_PCV_ and RBC count from Coulter Counter), MCV_CC_ (direct measurment from Coulter Counter), and the MCV_CTV_ (calculated from sedimentation rate data) values for all 31 healthy blood samples.

The estimation of MCV (Eq. 8) calculated from the Hct obtained by the PCV method and the RBC count obtained from CC, gives an MCV range of 70–110 fL, which matches well with the literature values reported for healthy individuals^26^. However, measurements from the Coulter Counter and CTV, using Eq. (5), give us values around 50 fL. The close overlap of size distribution data from the CC and the CTV is shown in Fig. 2. It can be seen from the bar graph that MCV_ave_ is greater than MCV_CC_ and MCV_CTV_ for all 31 healthy donor samples.

**Figure 3:**
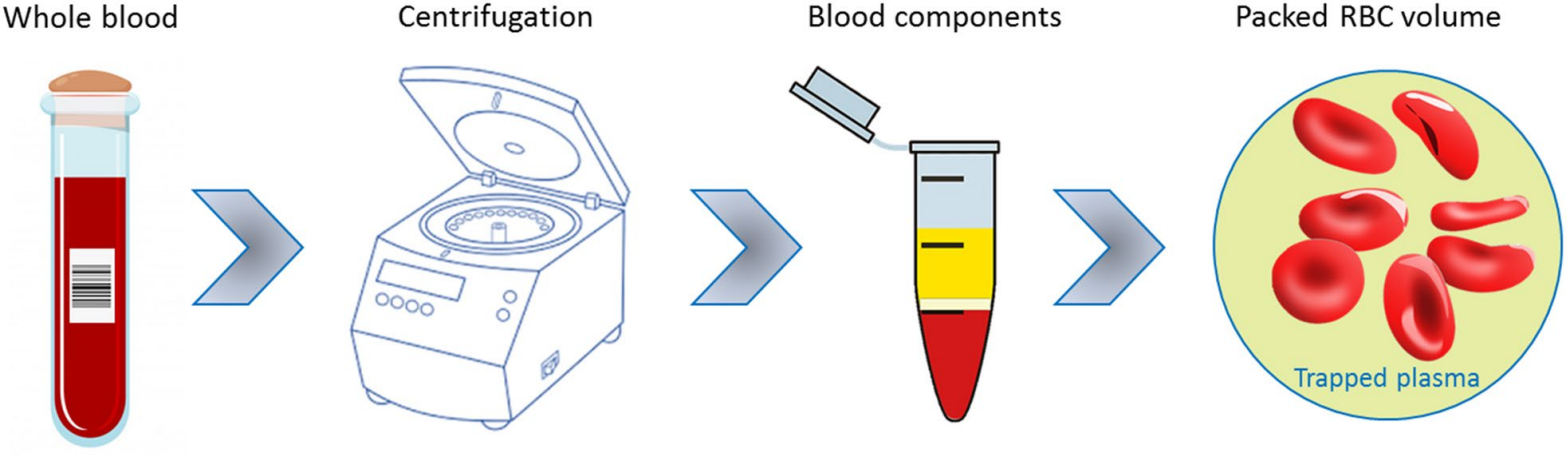
Schematic representation of plasma entrapment during Hct measurements by the PCV method.

As mentioned, for the determination of MCV_ave_ (Eq. 8), data obtained from PCV and CC are employed. Values from CC related to RBC count are within the normal range for healthy adults, as seen in Table 1^26^. Values of Hct obtained from PCV method are also within the normal range (around 45%). The disagreement between the MCV obtained by Eq. (8) and the value obtained from CC and CTV may be due, in part, to the fact that the PCV method uses centrifugal force to pack the RBCs in a hematocrit capillary tube, which inevitably suffers from plasma entrapment, illustrated in Fig. 3. Thus, the discrepancy in MCV between the different methods is partly attributed to the plasma entrapment when measuring Hct in this manner. Nevertheless, it has been reported that less than 5% of plasma is trapped in the packed RBC layer during centrifugation^26^. For example, Paterakis et al.^27^ have suggested that the trapped plasma constitutes less than 3% for any normal or abnormal samples, including oxygenated sickle cells.

Additionally, hematology analyzers routinely used to measure MCV such as the ADVIA treat RBCs with sodium dodecyl sulfate to make the cells spherical and glutaraldehyde to fix the membrane. Cell sizes are then measured using low angle and high angle light scattering while flowing in a single file order^28^. Although the Mie scattering theory requires objects to be spherical and altering the morphology from a disc to sphere does not necessarily alter the MCV, osmotic pressure changes in the carrier fluid of sodium dodecyl sulfate would alter the cell volume considerably and may be a source of MCV overestimation^29,30^.

Another plausible explanation for this disagreement is the fact that RBCs are deformable and some analyzers do not take into account the erythrocyte deformability. This could lead to an underestimation of the MCV measured on CC or CTV, as has been previously reported by others. For example, d’Onofrio et al.^31^ stated that electronic measures of MCV could be underestimated. In fact, these authors claim that electronic Hct_CC_ (measured on CC by using RBC count_CC_ and MCV_CC_) is a redundant parameter that could be abandoned, and that there are significant discrepancies between the manual Hct (obtained by PCV method), with the artifacts of plasma trapping and cell shrinkage, and the automated measurements. Thus, they suggest that calculation of Hct from the mean or the sum of pulse sizes and RBC count leads to systematic underestimation of automated Hct (and MCV) compared to the manual PCV measurement. Finally, Brugnara et al.^32^ reported that individual MCV measurements on different hematology analyzers are dependent on the technology used and that impedance-based instruments (such as CC) might underestimate MCV in hypochromic RBCs. The differences in the technologies employed along with the fact that such impedance-based instruments might not account for the deformability of RBCs^33^ could contribute to the disagreement on the MCV obtained with the different techniques. So far, no internationally accepted reference method has been published for measuring MCV^34^.

Due to the previous explanations, and most importantly, because there is no reference method to measure MCV, we conclude that the CTV could potentially be employed for measuring MCV along with other hematological parameters as long as reference values or correction factors are established. Even though RBC deformability might not be considered on CTV, the values obtained from both CTV and Coulter Counter are very similar, and Coulter Counter is already considered as an accurate instrument for measuring cell and particle volume distributions.

**Figure 4:**
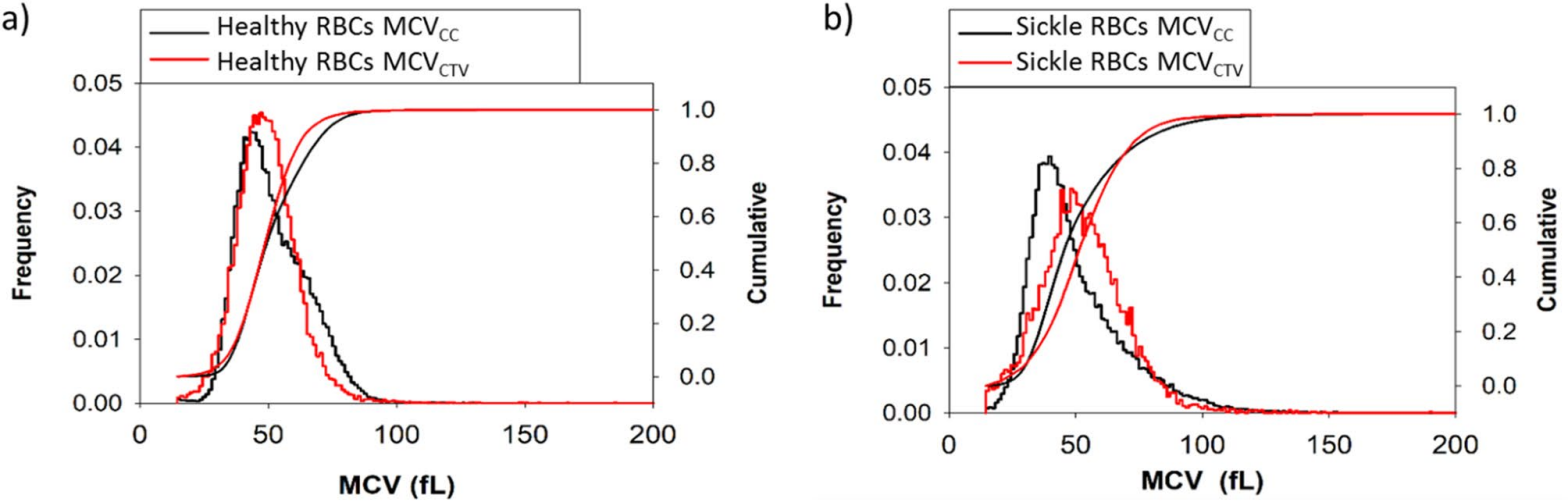
Volume histogram distribution (left y-axis) and cumulative curve (right y-axis) of RBCs measured on Coulter Counter (black) and CTV (red) for (**a**) all 31 healthy RBC samples and (**b**) all 15 sickle RBC samples.

A Coulter Counter calculates the volume of a cell by measuring the voltage difference between the inside and outside of an aperture tube (that is both filled with and submerged in an electrolyte solution) every time an object passes through the orifice of the tube and creates resistance in the circuit. The red cell volume distribution reported by the Coulter Counter, using electrical impedance, is in close agreement to the values reported by the CTV, which measures cell sedimentation to calculate volume. Figure 4a,b report the aggregate volume distributions of healthy and sickle RBC samples measured with both CC and CTV. These two methods show close agreement and high overlap between the histograms for both sets of subjects, yet CTV slightly underestimates MCV for healthy donors and overestimates for SCD subjects. The discrepancy in SCD data may be due to a number of factors including; increased density of sickle RBCs (due to dehydration), a lower sedimentation coefficient (due to sickling shape change), or an increased presence of large reticulocytes^35,36^.

Moreover, because of the disagreement in average MCV values between CC and CTV (51.8 and 49.6 fL) and the normal range (within 80–100 fL), and since RDW is calculated from the MCV, the values of RDW_CC_ and RDW_CTV_ are slightly higher than the normal value (generally below 15%). Specifically, RDW is obtained based on both the width of the cell volume distribution and the average cell size (i.e. it is calculated by dividing the standard deviation by the mean MCV). As can be seen in Table 1, the RDW values reported by CC and CTV are around 25%.

### Hemoglobin mass and concentration in individual RBCs

**Figure 5:**
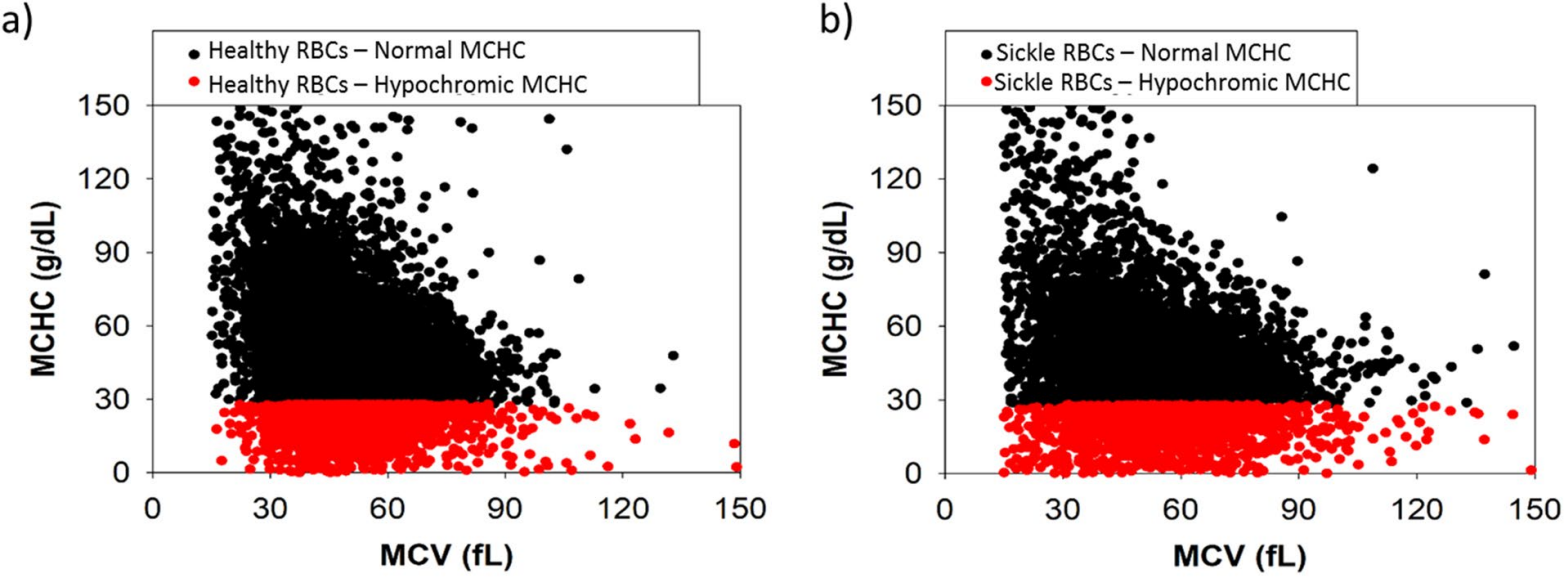
MCHC_CTV_ and MCV_CTV_ calculations for (**a**) all healthy RBCs and (**b**) sickle RBCs. Normal (black) cells and hypochromic (red) cells are shown with the cutoff value of 28 g/dL.

**Figure 6:**
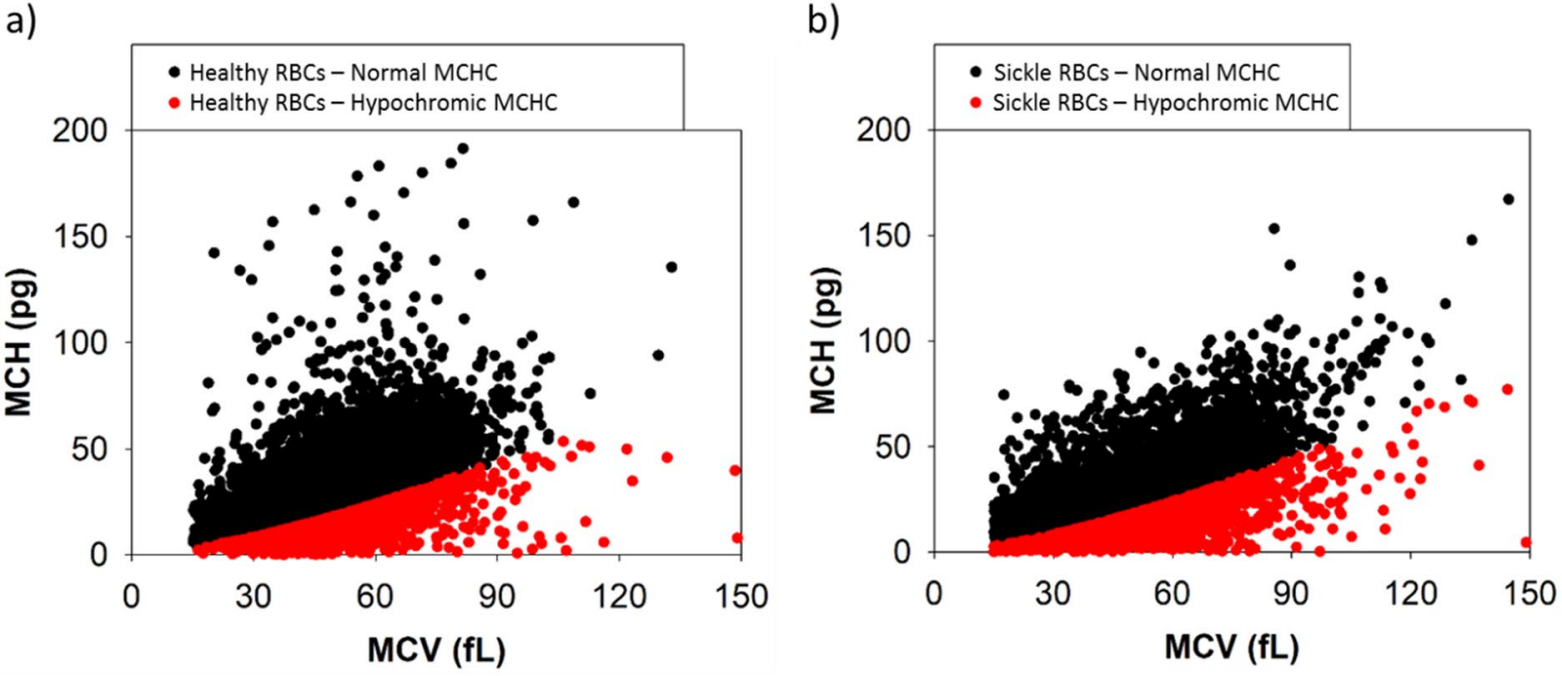
MCH_CTV_ and MCV_CTV_ calculations for (**a**) all healthy RBCs and (**b**) sickle RBCs. Normal (black) cells and hypochromic (red) cells are shown with the cutoff value of 28 g/dL.

The calculated MCHC_CTV_ scatterplots for all healthy donors and SCD subjects are plotted as a function of MCV_CTV_ in Fig. 5a,b. Black dots correspond to the RBCs above the hypochromic cutoff value of 28 g/dL and the red dots represent the subfraction of hypochromic RBCs. A high-volume hematology analyzer such as ADVIA uses light scattering and spectral absorption to determine MCHC while CTV measures induced velocity of RBCs that contain ferric Hb under a strong and constant magnetic energy gradient. Similarly, Fig. 6a,b plot MCH_CTV_ as a function of MCV_CTV_ for the same populations (healthy and sickle RBC samples, respectively) showing the MCHC hypochromic cutoff. Figures 5 and 6 demonstrate that our CTV system can measure the same parameters with detailed understanding of the population, as thousands of RBCs can be tracked per sample.

By observing both Fig. 6 and Table 1, it can be concluded that the average MCH (expressed in pg of Hb per RBC) reported by CTV is around the normal range reported for healthy samples (27–33 pg) and the global average for healthy RBC donors is 29.62 pg. Moreover, our previous study assesed the performance of CTV on measuring mass of Hb on individual RBCs by comparing CTV results to the standard method based on spectrophotometry, and our instrument reported accurate measurements and analyses^24^. Nevertheless, when comparing the MCHC measured by CTV to the reference range of 28–41 g/dL^26^, CTV measured higher MCHC because MCV_CTV_ is smaller than the reference range (see Table 1).

Pearson’s correlation coefficients (r) between CTV-measured MCHC/MCV and MCH/MCV for healthy and SCD samples were calculated for a total of 17,222 healthy and 7655 sickle RBC tracks. An r value of + 1.0 indicates a perfect positive correlation between two variables while an r value of − 1.0 indicates perfect negative correlation. Thus, r^2^ can be used to describe the correlation in either case. For the healthy RBC population, r_MCHC-MCV_ = − 0.249 and r_MCH-MCV_ = 0.442. For SCD samples, r_MCHC-MCV_ =  0.298 and r_MCH-MCV_ = 0.604. This represents the strength of fit between the intracellular iron content and its dependence on cell volume. The results suggest that larger cells have lower MCHC and higher MCH than smaller cells, and that the MCH dependence on volume is stronger than the trend of MCHC. Additionally, iron status in SCD subjects has a stronger dependence on volume than healthy ones; meaning, healthy individuals’ cells iron content is more independent of size than for those with SCD. The average values, standard deviation and coefficient of variation (CV) for MCV, MCHC and MCH in healthy and SCD populations are displayed in Table 2. Most notable from studying the variance is the wider distribution in MCV for SCD samples. CTV measured slightly higher iron content in healthy donors with similar variance between healthy and SCD samples. The small discrepancy between values reported in Table 1 may be due to the different number of cells measured between each donor. Analysis of variance and correlation between these parameters might be used to diagnose abnormal iron status in subjects. Provided a sufficiently large dataset for the subject, and baseline measurments of their gender, age, etc., CTV may be used to assess the snapshot of a subject’s iron status over the past 120 days (the approximate life of an RBC in vivo)^37^.

**Table 2:** Average, variance, standard deviation, coefficient of variation (CV) and Pearson’s correlation coefficients (r) with MCV_CTV_ of several iron status parameters for all tracked healthy and sickle RBCs.

	Mean	Variance	Std Dev	CV (%)	r_with MCV_CTV_
Healthy samples—MCV_CTV_ (fL)	49.6	131.6	11.5	23.1	
SCD samples—MCV_CTV_ (fL)	53.0	281.5	16.8	31.7	
Healthy samples—MCV_CC_ (fL)	52.3	180.2	13.4	25.7	
SCD samples—MCV_CC_ (fL)	50.9	357.7	18.9	37.2	
Healthy samples—MCHC_CTV_ (g/dL)	50.9	712.2	26.7	52.4	− 0.249
SCD samples—MCHC_CTV_ (g/dL)	44.1	661.3	25.7	58.3	− 0.298
Healthy samples—MCH_CTV_ (pg)	32.0	290.2	17.0	53.2	0.442
SCD samples—MCH_CTV_ (pg)	30.2	303.7	17.4	57.7	0.604

**Figure 7:**
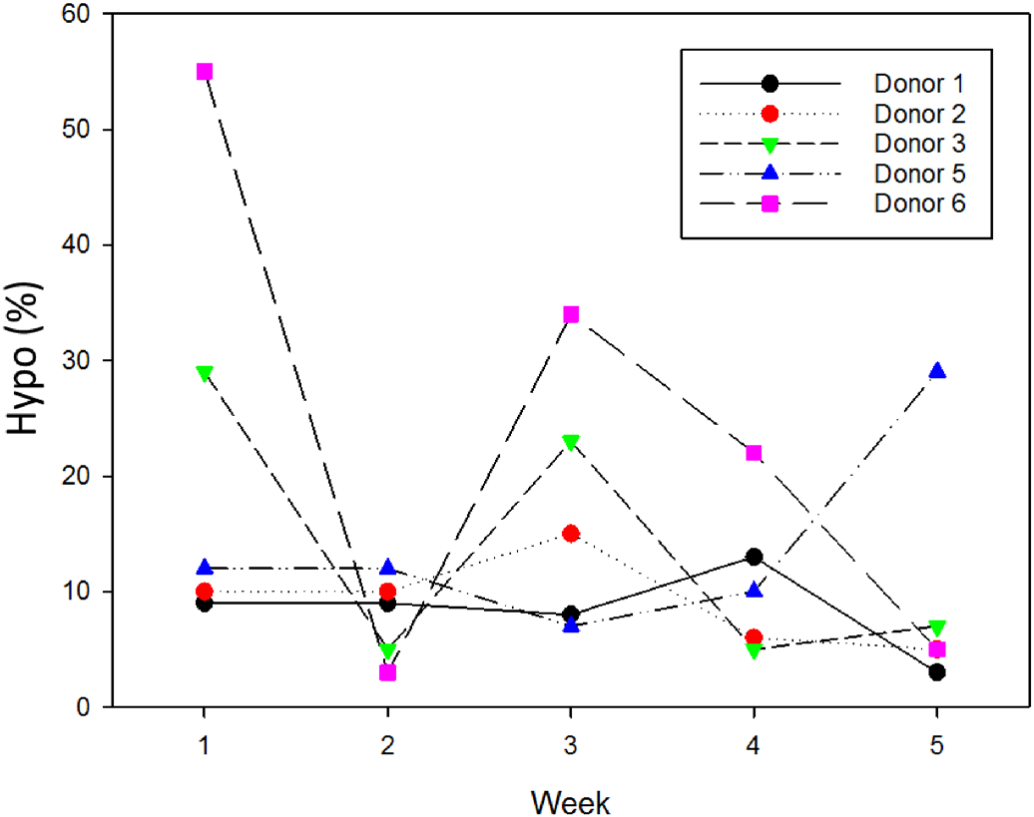
Temporal evolution of the percentage of hypochromic RBCs measured on CTV for all healthy donors.

Additionally, observing Table 1 also shows that besides MCH_CTV_, the overall Hb_spec_ of healthy donor 6 is much lower compared to other healthy donors, and the hypochromic fraction is much greater compared to the samples obtained from healthy donors 1–5. We have represented the temporal evolution of Hypo (%) for all the healthy donors (blood was drawn from each healthy donor on a weekly basis) in Fig. 7. It is known that during recombinant human erythropoietin (rHuEPO) therapy for anemia, Hypo > 10% is an indication for iron supplement requirement^16^. The fraction of hypochromic RBCs for healthy donor 6 is as high as 55%, and as low as 3%, over the 4-week testing period while other healthy donors’ values stay around the cutoff value. Hypo has been considered as a very sensitive marker because small changes in the number of RBCs with inadequate hemoglobin can be measured before there is any change in the MCHC^15^. Thus, quantification of hypochromic and/or hyperchromic red cells is helpful in the diagnosis of anemia. In fact, in a population of young anemic females, the percentage of hypochromic RBCs had the highest accuracy in distinguishing IDA from other anemias with normal iron stores^29,32^. Based on this, we concluded that our CTV can be very useful not only in measuring the mass of Hb in individual RBCs, but also on the diagnosis of the IDA.

**Figure 8:**
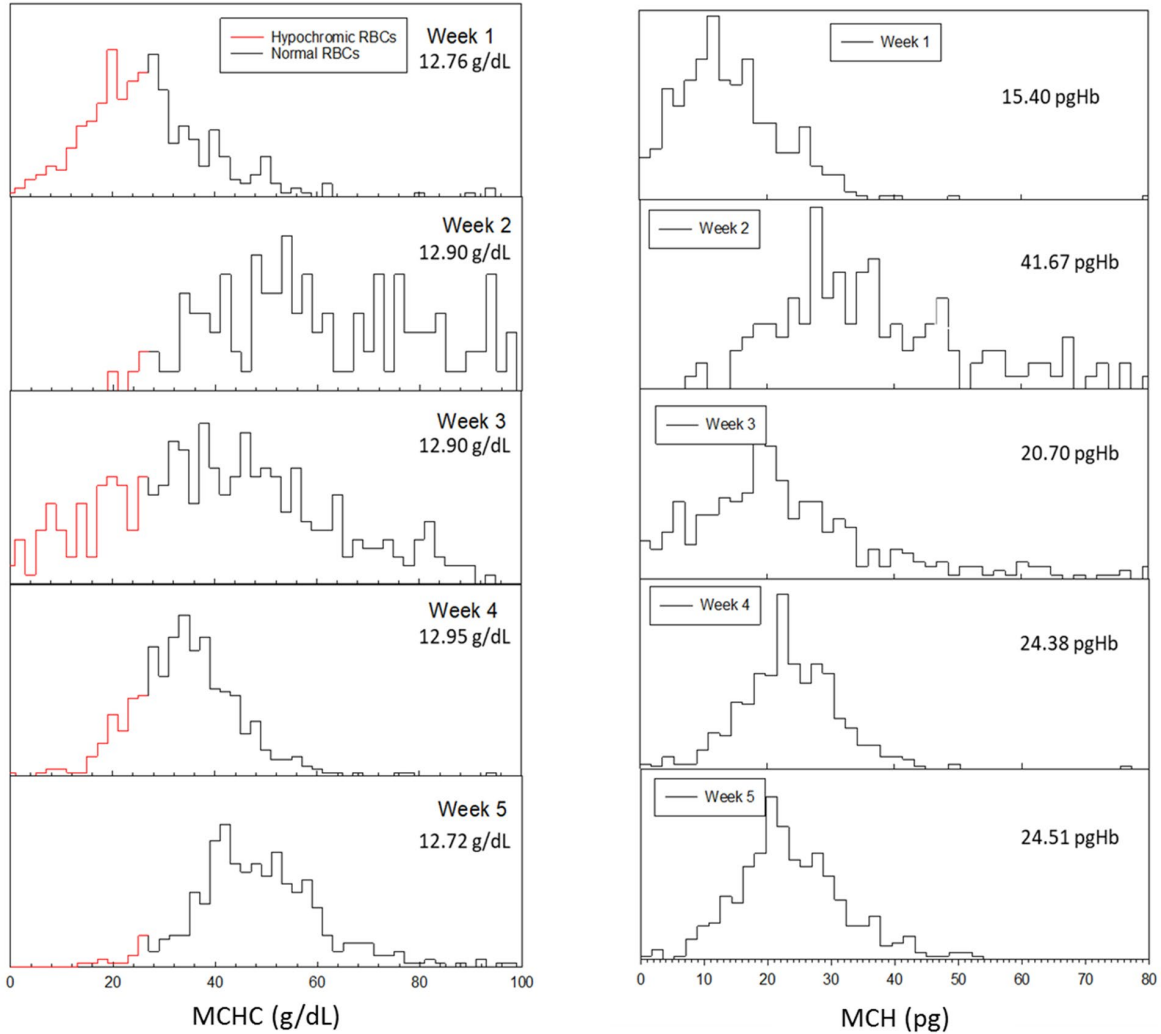
Weekly measurements of MCHC and MCH on CTV for healthy donor 6. Left panel presents MCHC_CTV_ (red line shows the hypochromic RBCs and the numbers inside the graph report Hb_spec_) and right panel shows MCH_CTV_ (numbers inside the graphs report the average MCH_CTV_).

Finally, and for further analysis on this healthy donor samples (donor 6), the histogram of the weekly measured MCHC and MCH values has been presented in Fig. 8. On the left side of Fig. 8, the red line corresponds to the hypochromic RBCs and the black line presents normal RBCs in terms of Hb concentration. As expected, the normal RBC population decreases as the Hypo percentage increases and vice versa. One interesting fact is that while the overall Hb concentration stays around 12.7–12.9 g/dL, the ratio of the hypochromic RBCs and normal RBCs seems to have a dynamic fluctuation. For comparison, the histogram of Hb mass is presented on the right side of Fig. 8, showing a similar fluctuation to the MCHC presented on the left side of Fig. 8.

## Conclusion

Over the last few years, several studies have emphasized the global concern about the prevalence of iron deficiency anemia (IDA) or its precursor state, iron deficiency, in both developed and developing countries. In fact, reducing anemia by 50% in women is a 2025 World Health Assembly Global Nutrition Target^38^. Detection of anemia in state-of-the-art hospitals and clinics can be easily performed through complete blood counts. However, most of hematology analyzers currently available are large instruments, are not economical or portable and often require trained technicians. Thus, there is a need for a portable and economical hematology analyzer that can be use at the point of need, such as at mobile blood donation platforms for screening purposes. For example, it has been stablished that the Hb of a blood donor drops by 1–1.5 g/dL after donating a single unit of whole blood^39^. Thus, an appropriate pre-donation test may mitigate the possibilities of rendering the blood donor anemic, which has also an explicit implication on the recipient’s health. However, no testing methodology and sample requirement have been specified for Hb screening^39^.

In this work, we evaluate the performance of a system referred to as the cell tracking velocimetry, CTV, to measure several hematological parameters from both fresh healthy human blood obtained from healthy donors and from blood obtained from SCD subjects. This system is based on the paramagnetic behavior that methemoglobin containing RBCs experience when suspended in water after applying a magnetic field. CTV uses a combination of magnets and microfluidics and has the ability to track the movement of thousands of red cells; thus, unlike other analyzers, CTV is able to measure not only traditional RBC indices but also novel parameters that are only available for analyzers that assess erythrocytes on a cell by cell basis. As such, we report for the first time the use of our CTV as a hematology analyzer able to measure MCV, MCHC, MCH, RDW or the percentage of hypochromic RBCs.

Our results indicate that most of the parameters measured with CTV are within the normal range for healthy adults, especially MCH. However, measures of the RBC volume (and thus, parameters related to volume such as MCHC and RDW) are outside the normal range for healthy samples; both CTV and CC reported MCV values that are almost half of the value for a normal adult. The reasons for these discrepancies are attributed in part to the plasma entrapment when measuring Hct (used to calculate the MCV by the traditional method) and also to the deformability of red cells, which is not taken into account when measuring MCV by CC and CTV. Since no internationally accepted reference method has been published for measuring MCV, we conclude that CTV could potentially be employed for measuring MCV along with other hematological parameters such as MCH, MCHC, RDW and hypochromic red cells, as long as instrument-specific reference ranges are stablished or correction factors are applied, as happens with several commercial analyzers. Moreover, the analysis of MCH and MCHC of SCD samples reported a stronger dependence on volume than healthy samples, which implies that the iron content of RBCs from SCD subjects is more dependent of cell size. Also, we observed a wider distribution in MCV for SCD samples. Future technology and methodology improvements may enable the direct measurement of RBC density, leading to more accurate estimation of hematology parameters and future works that analyze the density population characteristics.

While this work presents the potential of CTV to be used as point of care device for anemia detection, it should be noted that our benchtop CTV makes use of a microscope/camera that hinders its use at the point of care. The focus of our future work is replacing the microscope and camera system by a smartphone, laptop, or portable camera, to gain portability. Comparing the resolution and accuracy of our system to other hematology analyzers, along with its potential portability, price and fast measurement (less than 5 min for tracking thousands of metHb-RBCs), we believe that CTV is a promising technology to be used for blood testing at the point of care or in low resource areas. Potential applications include: population screening for anemia, assessing the eligibility of blood donors, triage of trauma victims, and perioperative assessment of a subject’s transfusion needs, which will facilitate real-time clinical decision making.

## Supplementary Information

Supplementary Information.
