# Supplementary material for: Potential of cell tracking velocimetry as an economical and portable hematology analyzer
Source: Sci Rep. 2022 Feb 1;12:1692. doi: 10.1038/s41598-022-05654-5 (PMC8807587; doi:10.1038/s41598-022-05654-5)
Supplement: Supplementary file 1 — Supplementary Information. [file 41598_2022_5654_MOESM1_ESM.docx]

**Supplementary information**

**Potential of Cell Tracking Velocimetry as an Economical and Portable Hematology Analyzer**

Jenifer Gómez-Pastora^a^, Mitchell Weigand^a^, James Kim^a^, Andre F. Palmer^a^, Mark Yazer^b^, Payal C. Desai^c^, Maciej Zborowski^d^, Jeffrey J. Chalmers^a^

^a^William G. Lowrie Department of Chemical and Biomolecular Engineering, The Ohio State University

151 West Woodruff Avenue, Columbus, OH 43210

^b^Department of Pathology, University of Pittsburgh, 3636 Blvd of the Allies, Pittsburgh, PA 15213

^c^Department of Internal Medicine, Division of Hematology, The Ohio State University, Lincoln Tower - 1100-D, 1800 Cannon Drive, Columbus, OH 43210

^d^Department of Biomedical Engineering, Cleveland Clinic, 9500 Euclid Avenue, Cleveland, OH 44195

**Table of contents**

Figure S1. Working principle of CTV.

Figure S2. Optimization of the CTV operation procedure.


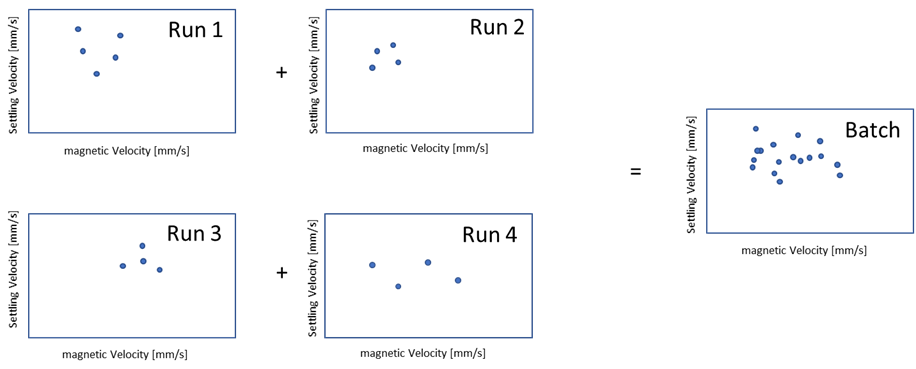


Figure S1. Working principle of CTV. CTV uses a combination of a microscopic camera and a magnetic microfluidic channel to capture the movement of biological samples introduced into the system under a constant magnetic flux. The camera captures multiple images of the samples’ movement in series so that the image processor can calculate the magnetic and settling velocity based on the trajectory. Approximately 50-100 trajectories (data points) are calculated each run (multiple images) and the data points from multiple runs are merged to constitute up to 1000 data points.


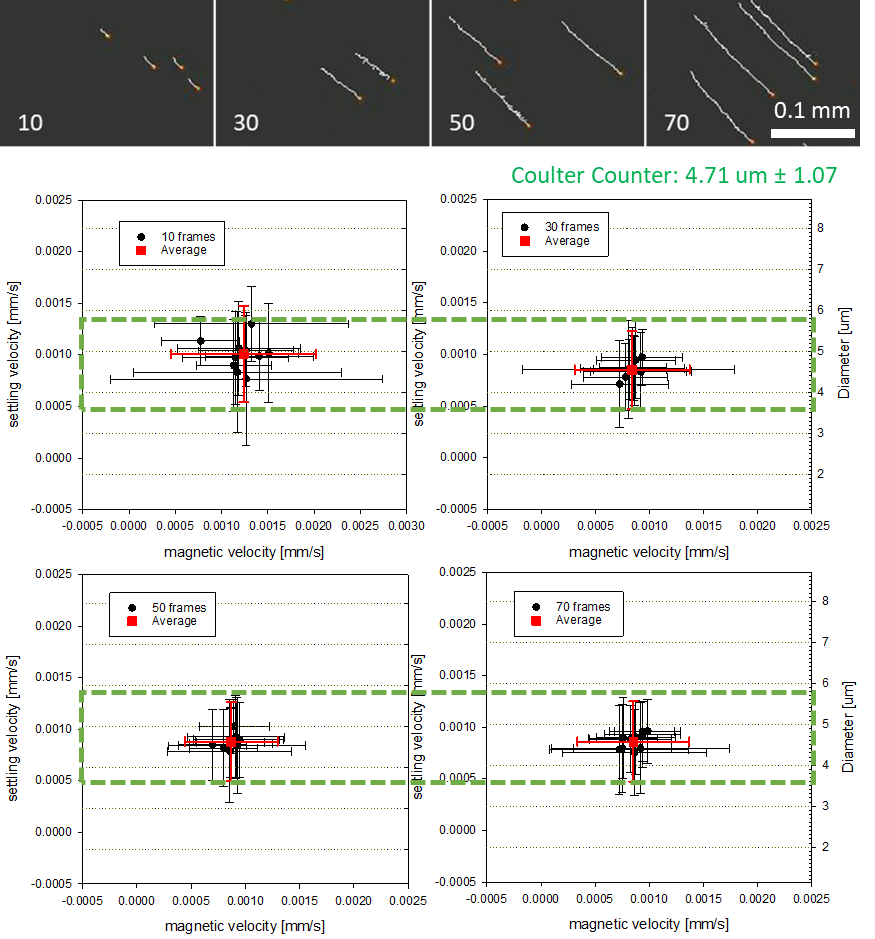


Figure S2. Optimization of the CTV operation procedure. Using the same protocol (with the exception of the number of images), 10 runs of 10, 30, 50, 70 images at 1 s interval were collected and the obtained magnetic/settling velocity was compared to each other, as well as the diameter distribution from Multisizer 4e Coulter Counter. The result from the data acquisition comparison study is presented here. As can be seen, 10 and 30 images are not enough to accurately calculate the trajectory of the RBCs, in contrast to 50 and 70, where the average and standard deviation of each samples are similar in distribution. The red dot plot is the average and standard deviation of the converged data set, and the green line represents the diameter distribution measured from the Multisizer 4e Coulter Counter.
